# Serial cfDNA assessment of response and resistance to EGFR-TKI for patients with EGFR-L858R mutant lung cancer from a prospective clinical trial

**DOI:** 10.1186/s13045-016-0316-8

**Published:** 2016-09-13

**Authors:** Qing Zhou, Jin-Ji Yang, Zhi-Hong Chen, Xu-Chao Zhang, Hong-Hong Yan, Chong-Rui Xu, Jian Su, Hua-Jun Chen, Hai-Yan Tu, Wen-Zhao Zhong, Xue-Ning Yang, Yi-Long Wu

**Affiliations:** Guangdong Lung Cancer Institute, Guangdong General Hospital & Guangdong Academy of Medical Sciences, 106 Zhongshan Er Road, Guangzhou, 510080 China

**Keywords:** Lung cancer, Plasma *EGFR* mutation, Quantitative change, Circulating free DNA, EGFR-TKI

## Abstract

**Background:**

Detecting epidermal growth factor receptor (*EGFR*) activating mutations in plasma could guide EGFR-tyrosine kinase inhibitor (EGFR-TKI) treatment for advanced non-small cell lung cancer (NSCLC). However, dynamic quantitative changes of plasma *EGFR* mutations during the whole course of EGFR-TKI treatment and its correlation with clinical outcomes were not determined. The aim of this study was to measure changes of plasma *EGFR* L858R mutation during EGFR-TKI treatment and to determine its correlation with the response and resistance to EGFR-TKI.

**Methods:**

This study was a pre-planned exploratory analysis of a randomized phase III trial conducted from 2009 to 2014 comparing erlotinib with gefitinib in advanced NSCLC harboring *EGFR* mutations in tumor (CTONG0901). Totally, 256 patients were enrolled in CTONG0901 and randomized to receive erlotinib or gefitinib. One hundred and eight patients harbored L858R mutation in their tumors and 80 patients provided serial blood samples as pre-planned scheduled. Serial plasma L858R was detected using quantitative polymerase chain reaction. Dynamic types of plasma L858R were analyzed using Ward’s hierarchical clustering method. Progression-free survival (PFS) and overall survival (OS) were compared between different types.

**Results:**

As a whole, the quantity of L858R decreased and reached the lowest level at the time of best response to EGFR-TKI. After the analysis of Ward’s hierarchical clustering method, two dynamic types were found. In 61 patients, L858R increased to its highest level when disease progressed (ascend type), while in 19 patients, L858R maintained a stable level when disease progressed (stable type). Median PFS was 11.1 months (95 % CI, 6.6–15.6) and 7.5 months (95 % CI, 1.4–13.6) in patients with ascend and stable types, respectively (*P* = 0.023). Median OS was 19.7 months (95 % CI, 16.5–22.9) and 16.0 months (95 % CI, 13.4–18.5), respectively (*P* = 0.050).

**Conclusions:**

This is the first report finding two different dynamic types of plasma L858R mutation during EGFR-TKI treatment based on a prospective randomized study. Different dynamic types were correlated with benefits from EGFR-TKI. The impact of plasma L858R levels at disease progression on subsequent treatment strategy needs further exploration.

**Trial registration:**

ClinicalTrials.gov, NCT01024413

**Electronic supplementary material:**

The online version of this article (doi:10.1186/s13045-016-0316-8) contains supplementary material, which is available to authorized users.

## Background

Epidermal growth factor receptor (*EGFR*) activating mutations in the tyrosine kinase domain serve as predictive biomarkers for EGFR-tyrosine kinase inhibitor (EGFR-TKI) treatment outcome for patients with advanced non-small cell lung cancer (NSCLC) [1–6]. However, due to the invasive procedures required to obtain tumor tissues, not all patients can provide enough high-quality tissues for *EGFR* mutation analysis. Circulating free DNA (cfDNA) in plasma provides a noninvasive substitute for tumor tissues [7]. Several studies have reported a concordance rate between tumor and plasma >90 %, even reaching 97 %, demonstrating the feasibility of detecting *EGFR* mutations in cfDNA [8–10]. *EGFR* mutation status detection in cfDNA has been approved by the European Society for Medical Oncology and by China to be used with EGFR-TKI treatment for NSCLC [11, 12].

In addition to providing pretreatment information, plasma-based *EGFR* mutation detection makes it possible to monitor dynamic changes in this mutation during treatment. Several studies have reported a quantitative change in *EGFR* mutations during EGFR-TKI treatment by comparing pre- and post-treatment plasma, in which various types of plasma *EGFR* mutations were found [13–15]. The quantity of the plasma *EGFR* mutation sometimes decreases, or sometimes decreases slowly or rapidly. Patients whose plasma *EGFR* mutations decrease rapidly usually exhibit a better response to EGFR-TKI treatment [15]. However, these studies were not based on prospective clinical trials; therefore, the number of patients who had serial plasma specimens tested during EGFR-TKI treatments was limited, and very few plasma specimens were collected as part of a pre-planned schedule. The only recent study on plasma *EGFR* mutation changes based on a prospective clinical trial was reported by Mok et al. [16]. In this phase III trial (FASTACT-2), patients received gemcitabine/platinum plus sequential erlotinib or placebo. *EGFR* mutation-specific cfDNA levels decreased at cycle 3 and increased at the time of disease progression. Positive plasma *EGFR* mutant DNA at cycle 3 predicted a worse clinical outcome. In this study, the treatment was chemotherapy plus EGFR-TKI or placebo, not EGFR-TKI, and there was no information on the plasma *EGFR* mutation at other time points except at baseline, cycle 3, and at disease progression. The dynamic types of plasma *EGFR* mutations during the whole course of EGFR-TKI treatment and its correlation with clinical outcomes were not determined.

The present study was a pre-planned exploratory analysis of a randomized phase III trial comparing erlotinib with gefitinib treatment in advanced NSCLC patients containing *EGFR* mutations in tumor tissues (The Chinese Thoracic Oncology Group 0901, CTONG0901, NCT01024413). Serial plasma samples were collected as a pre-planned schedule, and the *EGFR* L858R mutation was detected using quantitative polymerase chain reaction (qPCR). We quantitatively measured changes in the L858R mutation and determined its correlation with clinical outcomes.

## Methods

### Study design and treatment

Serial plasma samples were taken from patients enrolled in CTONG0901, a single-center trial designed to compare erlotinib with gefitinib treatment in patients with advanced NSCLC who had *EGFR* activating mutations in tumor tissues. Eligible patients were over 18 years of age and had histologically or cytologically confirmed stage IIIB or IV NSCLC (AJCC/UICC, version 6) with *EGFR* activating mutations in their tumor samples, tested by direct sequencing as previously described [17]. Previously untreated patients and those receiving any type of systemic chemotherapy regimen without prior exposure to any EGFR-TKI were recruited. Eligible patients were 1:1 randomized to receive erlotinib (150 mg, po, qd) or gefitinib (250 mg, po, qd) until disease progression, unacceptable toxicity, or discontinuation of treatment due to other reasons. The primary endpoint was progression-free survival (PFS). Secondary endpoints included objective response rate (ORR), overall survival (OS), disease control rate (DCR), post-progression survival (PPS), safety, and biomarker analyses.

This trial was conducted in Guangdong Lung Cancer Institute of Guangdong General Hospital. It adhered to the Declaration of Helsinki and the Good Clinical Practice guidelines. The protocol was approved by the ethics committee at Guangdong General Hospital (committee’s reference number: GDREC [2009]011). All patients provided written informed consent for participation, with separate consent obtained for tumor specimens and/or blood samples for biomarker analyses. The pre-planned schedule for collecting serial blood samples during the course of EGFR-TKI treatment included baseline, 1 week after treatment, 1 month after treatment, and every 8 weeks until the appearance of disease progression. At each time point, 8 mL of blood was collected. Except for 1 week after treatment, other time points were exactly the same day when patients underwent computerized tomography (CT) scans for tumor response evaluation. Tumor response was evaluated according to the Response Evaluation Criteria in Solid Tumors (RECIST), version 1.1. The resistance was defined as disease progression according to RECIST, and the best response was the greatest reduction in tumor burden.

### Biomarker analyses

Blood samples were collected in tubes containing EDTA according to a fixed schedule. Plasma was immediately separated from blood cells by centrifugation at 3000 rpm at 4 °C for 5 min. Supernatants were collected and stored at −80 °C until assays were performed. cfDNA was isolated from 4 mL plasma using a QIAamp DNA blood mini kit (Qiagen, Hilden, Germany) according to the manufacturer’s instructions. To increase the testing sensitivity, we repeated the isolation five times and then pooled and concentrated the cfDNA from a total of 4 mL plasma for mutation testing.

We analyzed plasma only from patients containing the L858R mutation. Quantitative analyses of the L858R mutation and the endogenous reference gene *antitrypsin* in the cfDNA were performed by qPCR using the Roche LightCycler^®^ 480 real-time PCR system (Roche Life Science, Penzberg, Germany) under the following conditions: 95 °C for 10 min for 1 cycle followed by 95 °C for 15 s and 60 °C for 1 min for 50 cycles. An L858R target assay and human *antitrypsin* primers with the same amplification efficiency were developed by Applied Biosystems (Foster City, CA, USA). Sequences of the L858R mutation primers were as follows: forward primer, GCAGCATGTCAAGATCACAGATTT, reverse primer, CCTCCTTCTGCATGGTATTCTTTCT; MGB-probe, FAM-CAGTTTGGCCCGCCCA; *antitrypsin* forward primer, GACACCGAAGAGGCCAAGAA, reverse primer, GAAGATGTAATTCACCAGAGCAAAAA; and MGB-probe, FAM-TGTGTCTCTGTCAAGCTCCTTGAC. The qPCR mixture contained 5 μL 2× LightCycler^®^ 480 Probes Master (Roche Life Science), 0.25 μL primer mix, TaqMan^®^ Probe Assay (Applied Biosystems), 1.75 μL nuclease-free water, and 3 μL sample DNA or calibrator or H_2_O (for the no template control). Each sample was run in duplicate. The differences between replicates were lower than 2 cycle thresholds. If the result from one sample did not meet this criterion, the procedure was repeated. DNA from NCI-H1975 cells (harboring L858R mutation) was used as positive control. Human reference genomic DNA (catalog G1471, Promega Corporation, Madison, WI) was used as negative control. PCR grade H_2_O was the non-template control. Two wells of positive controls, two wells of negative control, and two wells of non-template control were included in every run. A mixture of plasma DNA with the L858R mutation was used for calibration. The level of the L858R mutation was normalized to that of the *antitrypsin* gene. The relative L858R copy number of each plasma sample was calculated as follows: *R* (L858R copy number) = 2^− [(Ct sample L858R − Ct sample antitrypsin) − (Ct CalibratorL858R − Ct calibrator antitrypsin)]^.

In plasma at the time of disease progression, we tested the exon 20 T790M mutation using a T790M mutation detection kit (Amoy Diagnostics, Xiamen, China) according to the principle of amplification refractory mutation system (ARMS) as previously described [17].

### Statistical analyses

The plasma L858R copy number is described as the median range. Ward’s hierarchical clustering method was used to categorize the dynamic types of L858R. Based upon the schedule of CT scans, we selected seven time points for Ward’s hierarchical clustering analyses: baseline, 1 week after baseline, 8 weeks before the best response, the best response, 8 weeks after the best response, 8 weeks before disease progression, and disease progression. If a patient progressed early on during treatment and there were not seven time points available for analysis, the available time points were put on corresponding points of the model and the missing time points were supplemented by expectation maximum to maintain the stability of Ward’s hierarchical clustering analyses. The entire population was classified into different groups according to Ward’s hierarchical clustering analyses. The quantities of L858R mutations between groups were compared by unequally spaced repeated measures design analysis of variance (ANOVA). The ratio of change of L858R quantity from baseline to disease progression (*r*) was calculated as (quantity of L858R at disease progression − quantity of L858R at baseline)/(quantity of L858R at baseline). Cut point of *r* to separate different groups was analyzed using maximally selected rank statistics by R software. Constituent ratio of *EGFR* T790M and two kinds of EGFR-TKIs between groups were compared by chi-square or continuity correction tests. PFS was defined as the time from the date of randomization to that of disease progression or of death from any cause. OS was measured from the date of randomization to the date of death from any cause. PPS was measured from the date of disease progression to the date of death from any cause. Survival was estimated using the Kaplan-Meier method and is expressed as a median value with a range and a two-sided 95 % confidence interval (CI). A two-sided log-rank test was used to compare survival between groups. The multivariate Cox proportional hazards regression model (alpha = 0.05) was used to evaluate independent predictive factors associated with PFS. A two-sided *P* value <.05 was considered statistically significant. SPSS version 17.0 (SPSS, Inc., Chicago, IL) software was used.

## Results

### Patient characteristics

**Fig. 1:**
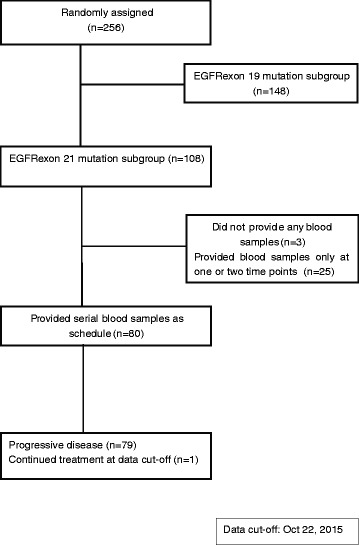
CONSORT diagram

From December 2009 to July 2014, a total of 256 patients were enrolled in CTONG0901. One hundred and eight patients harbored L858R mutation in their tumors tested by Sanger sequencing, and 105 patients provided blood samples, with 25 patients providing samples only at one or two time points and 80 patients providing serial blood samples as scheduled. The levels of plasma L858R were therefore tested in 80 patients. The study flow diagram is in Fig. 1. Their characteristics are summarized in Additional file 1: Table S1.

### Dynamic types of plasma L858R mutations

**Fig. 2:**
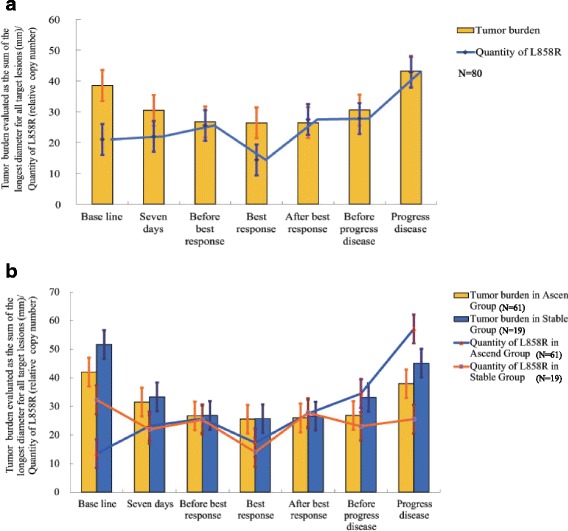
Dynamic change of plasma L858R mutation and tumor burden during EGFR-TKI treatment. **a** In total 80 patients, the quantity of L858R decreased to its lowest level at the time of best response to EGFR-TKI treatment, and then increased to its highest level when the disease progressed. **b** Using Ward’s hierarchical clustering analysis, 80 patients were classified into two groups according to their type of change. In one group, the quantity of L858R increased to its highest level at the time of disease progression (ascend group), while in the other group, the quantity of L858R did not increase and maintained a stable level as the disease progressed (stable group)

The L858R mutation was tested by qPCR in serial plasma samples from 80 patients at each time point, as scheduled. This mutation was detected in all plasma samples. As a whole, the quantity of plasma L858R decreased and reached the lowest level at the time of best response to EGFR-TKI treatment and then increased to its highest level when the disease progressed (Fig. 2a). By using Ward’s hierarchical clustering analysis, 80 patients were classified into two groups according to their type of change. The most dramatic difference between the two groups was, in one group, the quantity of L858R increased to its highest level at the time of disease progression (ascend type, group A, *n* = 61), while in the other group, the quantity of L858R did not increase and maintained a stable level as the disease progressed (stable type group S, *n* = 19) (Fig. 2b). Baseline characteristics of patients in two groups are shown in Table 1. ANOVA analyses showed that the effect of time was significant (*F* = 4.98, *P* < 0.01); the interaction of time and group was significant (*F* = 11.97, *P* < 0.01); over time, the quantity of plasma L858R copy number showed a linearly increasing trend (*F* = 23.97, *P* < 0.01); and the between-group test indicated that the variable group was not significant (*F* = 3.35, *P* = 0.071). The estimated cut point of *r* was 1.07 (*M* = 3.5877, *P* = 0.0063), which meant, compared with the quantity of L858R at baseline, the quantity at disease progression increased by more than 1.07 times which was the ascend type and increased by less than 1.07 times was the stable type. ANOVA analyses showed that the quantity of plasma L858R over time had no significant difference between patients receiving erlotinib and those receiving gefitinib (*F* = 0.160, *P* = 0.690). Furthermore, chi-square test showed that the constituent ratio of the ascend type and the stable type in patients who received erlotinib or gefitinib had no significant difference, either (*P* = 0.946).

**Table 1:** Baseline characteristics of patients in the ascend group and the stable group

Characteristics	Ascend group (*n*, %)	Stable group (*n*, %)	*P*
Gender			0.346
Male	30 (49.2)	7 (36.8)	
Female	31 (50.8)	12 (63.2)	
Age (median, range)	62 (40~84)	69 (40~84)	0.362
Body weight loss			1.000
<5 %	58 (95.1)	19 (100.0)	
≥5 %	3 (4.9)	0 (0.0)	
Smoking status			0.401
Never-smokers	41 (67.2)	15 (78.9)	
Smokers	20 (32.8)	4 (21.1)	
ECOG PS			1.000
0~1	58 (95.1)	18 (94.7)	
≥2	3 (4.9)	1 (5.3)	
Pathology			1.000
Adenocarcinoma	58 (95.1)	18 (94.7)	
Non-adenocarcinoma	3 (4.9)	1 (5.3)	
Clinical stage			1.000
IIIB	1 (1.6)	0 (0.0)	
IV	60 (98.4)	19 (100.0)	
Line of EGFR-TKI			0.371
First-line	36 (59.0)	9 (47.4)	
≥Second-line	25 (41.0)	10 (52.6)	
EGFR-TKI			0.946
Erlotinib	23 (37.7)	7 (36.8)	
Gefitinib	38 (62.3)	12 (63.2)	
Basic level of plasma L858R mutation (median, range)	20.0 (0.97~109.43)	27.45 (3.56~112.9)	0.184

*ECOG* Eastern Cooperative Oncology Group, *PS* performance status, *EGFR-TKI* epidermal growth factor receptor tyrosine kinase inhibitor

### Clinical outcomes

**Fig. 3:**
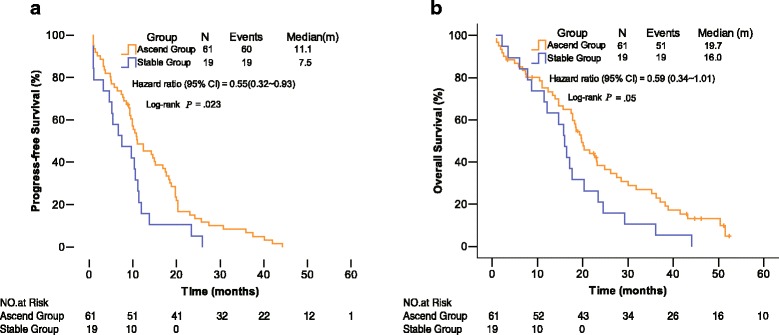
**a** Progression-free survival of patients in the ascend group and the stable group. **b** Overall survival of patients in the ascend group and the stable group

The cutoff date was October 22, 2015, and the median follow-up time was 20.7 months. In 80 patients, the PFS endpoint was observed in 79 patients (98.8 %), and 70 patients died (87.5 %). The median PFS was 10.4 months (95 % CI, 9.1–11.8), and the median OS was 18.6 months (95 % CI, 16.4–20.8) (Fig. 3). In group A, the median PFS was 11.1 months (95 % CI, 6.6–15.6), while in group S, it was 7.5 months (95 % CI, 1.4–13.6); the difference was statistically significant (HR = 0.55, 95 % CI, 0.32–0.93, *P* = 0.023) (Fig. 3a). Median OS showed a marginally statistical increase in group A (19.7 months, 95 % CI, 16.5–22.9 vs. 16.0 months, 95 % CI, 13.4–18.5, HR = 0.59, 95 % CI, 0.34–1.01, *P* = 0.050) (Fig. 3b). Median PPS was 5.5 months (95 % CI, 1.5~9.5) and 5.8 months (95 % CI, 2.9~8.6) (HR = 0.97, 95 % CI, 0.57–1.64, *P* = 0.898), respectively. The subsequent therapy was well-balanced between the two groups (Additional file 1: Table S2). There was no significant difference in median OS between groups when patients received subsequent best support care, or chemotherapy and/or local treatment. However, for patients who received subsequent other EGFR-TKIs (11 in group A, four in group S), the median OS differed significantly (38.2 months, 95 % CI, 8.9–67.5, vs. 15.8 months, 95 % CI, 2.3–29.2, *P* = 0.034) (Additional file 1: Table S3). The subsequent EGFR-TKIs included erlotinib, gefitinib, or icotinib which is a domestic first-generation EGFR-TKI in China. No patients received second- or third-generation EGFR-TKIs. The following variants were included in the multivariate Cox proportional hazards regression model: age (≥65, <65), pathology (adenocarcinoma, non-adenocarcinoma), weight loss (<5 %, ≥5 %), smoking status (never a smoker, smoker), family history of cancer (yes, no), Eastern Cooperative Oncology Group performance status (0–1, ≥2), line of EGFR-TKI therapy (first line, second line, or further), EGFR-TKI (gefitinib, erlotinib), and dynamic types of plasma L858R (group A, group S). The results showed that adenocarcinoma, PS 0–1, and group A were independent predictive factors associated with better PFS. Details are in Table 2.

**Table 2:** Variants associated with PFS in multivariate Cox proportional hazards regression model

Variables	*B*	SE	Wald	*P*	HR	95.0 % CI for HR
Pathology						
Non-adenocarcinoma					1.00	
Adenocarcinoma	−1.26	0.54	5.36	0.021	0.285	0.10~0.83
ECOG PS						
≥2					1.00	
0–1	−1.01	0.53	3.71	0.054	0.363	0.13~1.02
Groups						
Stable group					1.00	
Ascend group	−0.70	0.28	6.43	0.011	0.498	0.30~0.85

*PFS* progression-free survival, *ECOG* Eastern Cooperative Oncology Group, *PS* performance status

In 80 patients, 78 plasma samples at disease progression were tested for the T790M mutation and T790M was found in 17 samples, 11 samples in group A and six samples in group S (*P* = 0.305). There was no significant difference between patients with and without T790M in terms of PFS of EGFR-TKI treatment or post-progression survival (PPS) after EGFR-TKI treatment (Additional file 1: Table S4).

## Discussion

To our knowledge, this is the first report describing dynamic quantitative changes in plasma L858R amount during the whole course of EGFR-TKI treatment based on a prospective study. For the first time, we found that there were two different dynamic types of plasma L858R mutation during EGFR-TKI treatment. In the ascend type, the quantity of the L858R mutation increased to its highest level at disease progression, while in the stable type, the quantity of the L858R mutation did not increase and maintained a stable level at disease progression. Compared with the quantity of L858R at baseline, the quantity at disease progression increased by more than one time was the criteria for separating the two groups. The ascend type was associated with more therapeutic benefit from EGFR-TKI treatment than the stable type. Our results show, for the first time, that the entire L858R mutation population can be divided into two subtypes according to quantitative changes of the plasma L858R mutation.

In the present study, patients with different dynamic types achieved different benefits from EGFR-TKI treatment. However, the change of quantity of L858R mutation in cfDNA was not a useful predictive biomarker along the course of EGFR-TKI treatment because the difference of L858R mutation level was found until disease progression. Therefore, from this result, monitoring the dynamic change of *EGFR* activating mutation during EGFR-TKI treatment had low clinical predictive value. Two different dynamic types found in this study demonstrated that the *EGFR* mutation positive population could be divided into two subtypes. These two subtypes were correlated with different benefit from EGFR-TKI treatment and showed different dynamic characteristics of plasma *EGFR* activating mutation when disease progressed. The findings warrant further studies to explore the molecular mechanisms for the change of *EGFR* mutation in plasma. Genomic information from next-generation sequencing (NGS) might contribute to find the changes of predominant clones and the changes of other relative genes when disease progressed. Furthermore, the level of plasma *EGFR* mutation at disease progression might potentially influence subsequent treatment strategy. Our subsequent treatment analyses showed that patients with high levels of plasma L858R at disease progression achieved greater benefit from subsequent other first generation EGFR-TKIs than those with low levels of the L858R mutation. Better subsequent strategy might be made by combining the level of plasma *EGFR* activating mutation and resistant genetic information when disease progressed.

The *EGFR* exon 19 deletion and the exon 21 L858R mutations are the most common activating mutations. Patients whose tumors are characterized by these two mutations can achieve dramatic benefits from EGFR-TKI treatment. They are therefore considered typical activating mutations. However, an increasing number of studies have reported that two subtypes of patients achieve different benefits from EGFR-TKI treatment [18–20]. The clinical trial CTONG0901, on which our exploratory study was based, started in 2009 and it was originally designed to compare the efficacy of gefitinib or erlotinib treatment in patients only harboring the *EGFR* L858R mutation. After 6 months, the protocol was amended to enroll patients with either exon 19 deletion or exon 21 L858R mutation (Additional file 2). Therefore, we separated the exploration for plasma L858R mutation from exon 19 deletion. The present study only focused on L858R mutation, and our future plan is to explore the plasma exon 19 deletion to validate the result of L858R mutation. Another reason why we focused on L858R mutation in this study was that exon 19 deletion was more complex to be quantitatively detected than exon 21 L858R. More sensitive method was needed to quantitatively detect exon 19 deletion. In 2009 when the CTONG0901 trial was initiated, we used the qPCR for quantitative analyses of plasma L858R mutations. The sensitivity of qPCR for reliable mutational analysis is minimum 1~5 % of mutant alleles in a wild-type background [21–23]. Enough cfDNA from enrolled advanced NSCLC patients harboring relatively high abundance *EGFR* mutation in their tumors contributed to the high sensitivity in this study [17]. In the future, other testing methods, for example, droplet digital PCR or next generation sequencing, should be considered.

There are some limitations to the present study. We only detected T790M in the plasma at the time of disease progression by ARMS, which was the most commonly used method for detecting *EGFR* mutation when the study was designed. The sensitivity of ARMS detecting T790M was relatively low and only 17 out of 78 patients (22 %) were found to be T790M positive. We tried to detect the quantity of T790M mutation by qPCR during EGFR-TKI treatment but failed, and we could not do droplet digital PCR or beaming digital PCR at that time, which is more sensitive than qPCR or ARMS in detecting T790M [24, 25]. More T790M-positive patients found by more sensitive methods and the dynamic combination of activating mutation and T790M mutation could provide more information to predict the benefits of EGFR-TKI treatment and to guide subsequent application of third-generation EGFR-TKI which target both *EGFR* activating mutation and T790M mutation. Another limitation was, although CTONG0901 was a randomized trial, this exploratory research was based on patients who provided serial blood samples as scheduled. Therefore, there must be some selection bias which potentially impacted the results. The conclusion needs further validation.

## Conclusions

In summary, our study suggests that the L858R mutation population can be divided into two subtypes according to changes in the plasma L858R mutation. Different dynamic types were correlated with different benefits from EGFR-TKI treatment. Because the difference of L858R mutation level was found until disease progression, monitoring the dynamic change of L858R mutation during EGFR-TKI treatment had low clinical predictive value. The molecular mechanisms for the two subtypes and the impact of plasma L858R level at disease progression on subsequent treatment strategy needs further exploration. The optimal strategies to overcome EGFR-TKI resistance by combining the level of plasma *EGFR* activating mutation and resistant genetic information when disease progressed warrant further investigation.

## Additional files

Additional file 1: Table S1. Demographics and clinical characteristics of all patients. **Table S2.** Subsequent therapy after EGFR-TKI of two groups. **Table S3.** Survival comparison between two groups receiving different subsequent treatment after EGFR-TKI. **Table S4.** PFS and PPS between patients whose plasma at time of disease progression were with or without T790M. (DOC 119 kb) Additional file 2: Trial protocol. (DOCX 179 kb)

## Abbreviations

cfDNA: Circulating free DNA
CR: Complete response
CT: Computerized tomography
CTONG0901: The Chinese Thoracic Oncology Group 0901
DCR: Disease control rate
ECOG: Eastern Cooperative Oncology Group
EGFR: Epidermal growth factor receptor
EGFR-TKI: EGFR-tyrosine kinase inhibitor
NSCLC: Non-small cell lung cancer
ORR: Objective response rate
OS: Overall survival
PD: Progressive disease
PFS: Progression-free survival
PPS: Post-progression survival
PR: Partial response
PS: Performance status
qPCR: Quantitative polymerase chain reaction
RECIST: Response Evaluation Criteria in Solid Tumors
SD: Stable disease
