# Supplementary material for: Serial cfDNA assessment of response and resistance to EGFR-TKI for patients with EGFR-L858R mutant lung cancer from a prospective clinical trial
Source: J Hematol Oncol. 2016 Sep 13;9(1):86. doi: 10.1186/s13045-016-0316-8 (PMC5020532; doi:10.1186/s13045-016-0316-8)
Supplement: Additional file 1: Table S1. — Demographics and clinical characteristics of all patients. Table S2. Subsequent therapy after EGFR-TKI of two groups. Table S3. Survival comparison between two groups receiving different subsequent treatment after EGFR-TKI. Table S4. PFS and PPS between patients whose plasma at time of disease progression were with or without T790M. (DOC 119 kb) [file 13045_2016_316_MOESM1_ESM.doc]

**Additional file 1**

**Table S1** Demographics and clinical characteristics of all patients

|  | ***N*** | **%** |
| --- | --- | --- |
| Gender |  |  |
| Male | 37 | 46.3 |
| Female | 43 | 53.8 |
| Age (median, range) | 64.0(40.0~84.0) | |
| Smoking status |  |  |
| Never-smokers | 56 | 70.0 |
| Smokers | 24 | 30.0 |
| ECOG PS |  |  |
| 0-1 | 79 | 98.7 |
| 2 | 1 | 1.3 |
| Pathology |  |  |
| adenocarcinoma | 77 | 96.2 |
| non-adenocarcinoma | 3 | 3.8 |
| Clinical stage |  |  |
| IIIB | 1 | 1.3 |
| IV | 79 | 98.7 |
| EGFR-TKI |  |  |
| erlotinib | 30 | 37.5 |
| gefitinib | 50 | 62.5 |
| Line of EGFR-TKI |  |  |
| First-line | 55 | 68.8 |
| Second-line | 25 | 31.2 |
| Subsequent therapy |  |  |
| Best support care | 34 | 42.5 |
| Chemotherapy +/- local treatment^a^ | 16 | 20.0 |
| Other EGFR-TKIs | 15 | 18.8 |
| Other EGFR-TKIs + local treatment | 5 | 6.2 |
| Local treatment | 9 | 11.2 |
| Remain on EGFR-TKI without PD | 1 | 1.3 |
| Total | 80 | 100.0 |

*ECOG* Eastern Cooperative Oncology Group, *PS* performance status, *EGFR-TKI* epidermal growth factor receptor tyrosine kinase inhibitor, *CR* complete response, *PR* partial response, *SD* stable disease, *PD* progressive disease

^a^Local treatments included radiotherapy, interventional therapy, or surgery.

**Table S2** Subsequent therapy after EGFR-TKI of two groups

| Group | Subsequent therapy | | | | | *P* |
| --- | --- | --- | --- | --- | --- | --- |
|  | Best support care | Chemotherapy +/- local treatment | Other targeted therapy | Other targeted therapy + local treatment | Local treatment |  |
| Ascend Group | 27(45.0％) | 11(18.3％) | 11(18.3％) | 4(6.7％) | 7(11.7％) | 0.939 |
| Stable Group | 7(36.8％) | 5(26.3％) | 4(21.1％) | 1(5.3％) | 2(10.5％) |  |
| Total | 34(43.0％) | 16(20.3％) | 15(19.0％) | 5(6.3％) | 9(11.4％) |  |

*EGFR-TKI* epidermal growth factor receptor tyrosine kinase inhibitor

**Table S3** Survival comparison between two groups receiving different subsequent treatment after EGFR-TKI

| Best support care | N | Events | Median | | *P* |  |
| --- | --- | --- | --- | --- | --- | --- |
|  |  |  | Estimate | 95%CI |  |  |
| OS |  |  |  |  | 0.795 |  |
| Ascend Group | 34 | 31 | 17.7 | 10.7~24.7 |  |  |
| Stable Group | 9 | 9 | 14.6 | 7.3~21.9 |  |  |
| PPS |  |  |  |  | 0.750 |  |
| Ascend Group | 34 | 31 | 1.2 | 0.2~2.3 |  |  |
| Stable Group | 9 | 9 | 3.5 | 2.9~4.2 |  |  |
| Chemotherapy +/- local treatment | N | Events | Median | | *P* | |
|  |  |  | Estimate | 95%CI |  |  |
| OS |  |  |  |  | 0.174 | |
| Ascend Group | 11 | 10 | 30.0 | 17.3~42.7 |  | |
| Stable Group | 5 | 5 | 16.4 | 5.8~27.0 |  | |
| PPS |  |  |  |  | 0.804 | |
| Ascend Group | 11 | 10 | 11.6 | 8.0~15.2 |  | |
| Stable Group | 5 | 5 | 5.8 | 1.8~9.5 |  | |
| Other targeted therapy | N | Events | Median | | *P* | |
|  |  |  | Estimate | 95%CI |  |  |
| OS |  |  |  |  | 0.034 | |
| Ascend Group | 11 | 7 | 38.2 | 8.9~67.5 |  | |
| Stable Group | 4 | 4 | 15.8 | 2.3~29.2 |  | |
| PPS |  |  |  |  | 0.287 | |
| Ascend Group | 11 | 7 | 15.4 | 0.0~33.3 |  | |
| Stable Group | 4 | 4 | 6.0 | 12.3 |  | |
| Other targeted therapy + local treatment | N | Events | Median | | *P* | |
|  |  |  | Estimate | 95%CI |  |  |
| OS |  |  |  |  | 0.219 | |
| Ascend Group | 4 | 3 | 17.8 | 9.4~26.2 |  | |
| Stable Group | 1 | 1 | ---- | ---- |  | |
| PPS |  |  |  |  | 0.182 | |
| Ascend Group | 4 | 3 | 8.5 | 4.7~12.2 |  | |
| Stable Group | 1 | 1 | 24.7 | -- |  | |

*EGFR-TKI* epidermal growth factor receptor tyrosine kinase inhibitor, *OS* overall survival, *PPS* post-progression survival

**Table S4** PFS and PPS between patients whose plasma at time of disease progression were with or without T790M

|  | N | Events | Median | | *P* |
| --- | --- | --- | --- | --- | --- |
|  |  |  | Estimate | 95%CI |  |
| PFS |  |  |  |  |  |
| Without T790M | 61 | 60 | 10.4 | 9.0～11.8 | 0.112 |
| With T790M | 17 | 17 | 10.1 | 3.8～16.3 |  |
| PPS | N | Events | Median | | *P* |
|  |  |  | Estimate | 95%CI |  |
| Without T790M | 61 | 51 | 6.1 | 1.5～10.8 | 0.208 |
| With T790M | 17 | 17 | 3.3 | 0.0～7.2 |  |

*PFS* progression-free survival, *PPS* post-progression survival
