# Supplementary material for: Serial cfDNA assessment of response and resistance to EGFR-TKI for patients with EGFR-L858R mutant lung cancer from a prospective clinical trial
Source: J Hematol Oncol. 2016 Sep 13;9(1):86. doi: 10.1186/s13045-016-0316-8 (PMC5020532; doi:10.1186/s13045-016-0316-8)
Supplement: Additional file 2: — Trial protocol. (DOCX 179 kb) [file 13045_2016_316_MOESM2_ESM.docx]

**Clinical Trial Protocol**

| **Title** | **A randomized, controlled phase II/III trial to evaluate the efficacy of elortinib vs gefitinib in advanced non-small-cell lung cancer with EGFR exon 19 or 21 mutations** |
| --- | --- |
| **Protocol ID** | **CTONG0901** |
| **Principal Investigator** | **Dr. Yi-Long Wu** |
| **Study Site** | **Guangdong General Hospital Cancer Center** |
| **Contact Information** | **Address: Guangdong General Hospital Cancer Center, 106 Zhongshan 2^nd^ Road, Guanghzou (510080)**  **Tel: +8620, 83877557**  **Fax: +8620, 83800372**  **E-mail: syylwu@live.cn** |

# Study Design

**Part 1 (Phase II)**

Patients with advanced NSCLC that has not been treated or has progressed despite anti-cancer therapy

Collect tissue specimens for EGFR test

No mutation or exon19 mutation

Exclusion

Randomization

Elorinib 150mg qd

Efficacy and survival follow-up

Exon21 mutation

Gefitinib 250mg qd

**Part 2 (Phase III)**

Advanced NSCLC patients with histologically confirmed EGFR

exon19 or exon21 mutations

exon19 mutation

(newly enrolled)

Elorinib150mg qd

Randomization

exon21 mutation

(extensively enrolled)

Gefitinib 250mg qd

Efficacy and survival follow-up

**Table of Contents**

[Study Design 2](#_Toc437617682)

[1. Background 6](#_Toc437617683)

[2. Objectives 8](#_Toc437617684)

[3. Methodology 8](#_Toc437617685)

[3.1 Estimating sample size of Part 1, a randomized, controlled phase II clinical trial 8](#_Toc437617686)

[3.2 Estimating sample size of Part 2, a randomized, controlled phase III clinical trial 9](#_Toc437617687)

[4. Duration and Progress 9](#_Toc437617688)

[4.1 Enrollment time 9](#_Toc437617689)

[4.2 Follow-up time 9](#_Toc437617690)

[5. Selection of Subjects and Treatment of Noncompliance 9](#_Toc437617691)

[5.1 Target population 9](#_Toc437617692)

[5.2 Inclusion criteria 9](#_Toc437617693)

[5.3 Exclusion criteria 10](#_Toc437617694)

[5.4 Drop-out criteria 10](#_Toc437617695)

[5.5 Withdrawal criteria 10](#_Toc437617696)

[5.6 Treatment of noncompliance and dropouts 10](#_Toc437617697)

[6. Endpoints 11](#_Toc437617698)

[6.1 Primary endpoints 11](#_Toc437617699)

[6.2 Secondary endpoints 11](#_Toc437617700)

[6.3 Exploration endpoints 11](#_Toc437617701)

[7. Study Procedure 11](#_Toc437617702)

[7.1 Subject screening and baseline measurements 12](#_Toc437617703)

[7.2 Therapeutic regimens 12](#_Toc437617704)

[7.3 Follow-up procedure 14](#_Toc437617705)

[8. Efficacy and Safety Evaluations 15](#_Toc437617706)

[8.1 Efficacy assessment 15](#_Toc437617707)

[8.2 Adverse reactions 15](#_Toc437617708)

[8.3 PS score 15](#_Toc437617709)

[9. Ethical Requirements 15](#_Toc437617710)

[10. Study Quality Control 16](#_Toc437617711)

[11. Data Acquisition and Analytical Methods 16](#_Toc437617712)

[11.1 Data acquisition 16](#_Toc437617713)

[11.2 Statistical analysis 16](#_Toc437617714)

[11.3 Mid-term analysis 17](#_Toc437617715)

[References 17](#_Toc437617716)

# 1. Background

Epidermal growth factor receptor (EGFR) tyrosine kinase inhibitor (TKI) plays important roles in treatment of non-small cell lung cancer (NSCLC), however, its efficacy varies greatly in different populations. Benefit populations including women, adenocarcinoma patients and non-smokers are thought to be favorable whereas some non-benefit population may also have survival benefits from TKI treatment as well. Selecting effective patients to receive TKI therapy by biomarkers has been always an attractive topic of this field.

EGFR mutations are commonly found in non-smokers, women, Asian, and adenocarcinoma patients^1-4^ who have high remission rate of TKI therapy clinically.^5,6^ Numerous retrospective studies have demonstrated that clinical characteristics are associated with TKI efficacy and EGFR mutations. Among the Asian patient population, the mutation frequency accounts for 25%~50%, higher than that in Northern American and Western Europe (10%). One of the potential reasons is likely due to diverse genetic background. The remission rate to TKI treatment in EGFR-mutated patients is approximately 77%, compared with 10% in mutation negative patients. In addition, a number of studies have shown that EGFR-mutated patients have survival benefits from TKI therapy. MST is reported up to 30 months. In IDEAL trial, 46% of patients with mutations responded to TKI treatment compared to 10% of patients with wild-type EGFR (P=0.005), however, no survival benefit was observed.^7^ As of September 2008, the preliminary results of IPASS trial in which 1212 non-smoking or mildly smoking Asian patients with advanced lung adenocarcinoma received either first-line gefitinib monotherapy or first-line paclitaxel/carboplatin chemotherapy have shown that among EGFR mutation-positive patients PFS was significantly longer in patients treated with geninitib. On the contrary, chemotherapy was associated with a significantly longer PFS among patients with wild-type EGFR (P<0.0001).^8^ The response to gefitinib achieved 71.2% in EGFR mutation-positive patients while only 1.1% of patients with wild-type EGFR responded to gefitinib.^8^

Different EGFR mutations may be different as well. Further studies found that different mutations are associated with clinical outcomes of patients, e.g. a deletion in exon19 responded better to gefitinib and erlotinib than a point mutation (L585R) in exon21.^9,10^ (Figure 1)


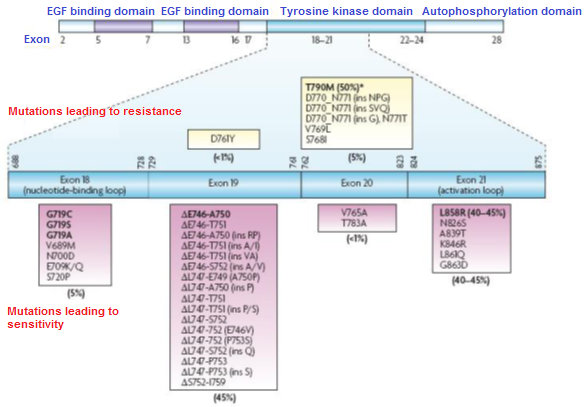


**Figure 1** EGFR mutation sites and their association with drug sensitivity

Rosell’s group in Spain reported a study at ASCO annual meeting in June 2009. The study that examined EGFR status in tumor tissues of 2507 advanced NSCLC patients found that 358 patients carried exon19 deletion or exon21 point mutation.^11^ In this study, the frequency of EGFR exon 19 or 21 mutations was 4.3% (358/2507). A total of 217 subjects were enrolled in this trial, including 62.3% of exon19 deletion and 37.7% of exon21 mutation. The patients with mutations were administrated with erlotinib. Among 197 patients with evaluable efficacy, the overall response was 70.6% (139/197). TTP was 14 months and the median survival time was 27 months.

In June 2008, an article published in JCO by Taiwan analyzed the outcomes of first-line gefitinib therapy in mutated patients. 106 patients were enrolled, 90 of whom provided specimens, including 20 cases of exon19 deletion and 23 cases of exon21 mutation. The remission rates were 95.0% and 73.9% and TTP accounted for 8.9 months and 9.1 months respectively. Other mutations were 16.7% and 2.3 months, and overall RR was 50.9%.^12^

In a study on gefitinib and control chemotherapy in first-line treatment of selective patients (IPASS) published in ASCO in June 2009 showed that the remission rate was 65% in patients with exon21 mutation.^13^

As EGFR inhibitors, erlotinib acts similarly as gefitinib by suppressing overexpressed EGFR TKI activity in solid tumors, however, both drugs vary greatly in pharmacokinetics. Oral administration of erlotinib 150 mg/d showed a higher plasma exposure, the resulting C_max_ and AUC_0-24h_ were comparable to those of gefitinib 700 mg/d; erlotinib has a shorter half-life than gefitinib so that repetitive dosing causes no drug accumulation. Recent studies found that the same dose of erlotinib (0.1g/kg/d) was superior to gefitinib in *in-vivo* model of H460A cell line with wild-type EGFR. In 2008, Costa *et al*’s study published in JCO showed that erlotinib inhibited mutated EGFR better than gefitinib in in-vitro studies. IC_50_ to L858R mutated cells was 0.04μM while IC_50_ of gefitinib was 0.08μM. In L858R-L747S mutated cells, IC_50_ of erlotinib was 0.08μM compared to 0.20μM of gefitinib.^14^ Collectively, insensitive patients with EGFR exon21 mutation may be favorable to erlotinib.

# 2. Objectives

**Primary Objectives:**

The objective of Part 1 is to verify that erlotinib can improve the remission rate (RR) of patients with EGFR exon21 mutation as compared with gefitinib.

The objective of Part 2 is to verity that erlotinib can prolong the progression-free survival (PFS) of patients with EGFR exon19 or exon 21 mutation as compared with gefitinib.

**Secondary Objectives:**

Erlotinib can prolong the overall survival of patients as compared with gefitinib.

Safety events of erlotinib and gefitinib in second-line population with EGFR exon19 or exon 21 mutation.

Pharmacoeconomic analysis of erlotinib and gefitinib in second-line population with EGFR exon19 or exon 21 mutation.

# 3. Methodology

## 3.1 Estimating sample size of Part 1, a randomized, controlled phase II clinical trial

Part 1 is a randomized, parallel-group phase II clinical trial that is intended to assess whether or not erlotinib can improve the efficacy in patients with exon21 mutation compared to gefitinib.

By using simple randomization and assuming that α=0.05, 1-β=0.8, the efficacy of gefitinib is 60%, the efficacy of erlotinib is enhanced by more than 15%, it is estimated to need 35 subjects per group to compare two independent proportions for a total of 70 subjects.

## 3.2 Estimating sample size of Part 2, a randomized, controlled phase III clinical trial

Part 2 is a randomized, parallel-group phase III clinical trial that is intended to verify that erlotinib can prolong the progression-free survival (PFS) of patients with EGFR exon19 or exon 21 mutation.

According to IPASS trial in 2009 and the data as described in “Screening for Epidermal Growth Factor Receptor Mutations in Lung Cancer” written by Rosell, it is assumed that the median PFS of EGFR mutation population who receives erlotinib and gefitinib are 9.5 months and 14.0 months, α=0.05, β=0.80, the duration of enrollment is 12 months, the duration of study is 48 months, the dropout rate is 5%, and both groups are randomized to enroll by 1:1, thus the sample size is estimated to be 254 subjects in total (127 subjects per group) by log rank tests using median survival time. Therein, patients with EGFR exon19 mutation are newly enrolled and those with EGFR exon21 mutation are enrolled during extension period of the phase II clinical trial.

# 4. Duration and Progress

## 4.1 Enrollment time

**Study progress**

Date when the first subject is expected to be enrolled: July 2009

Date when the last subject is expected to complete: July 2012

## 4.2 Follow-up time

2 years

# 5. Selection of Subjects and Treatment of Noncompliance

## 5.1 Target population

Advanced NSCLC patients with EGFR exon19 or exon21 mutation.

## 5.2 Inclusion criteria

1. Histologically or cytologically confirmed stage IIIB (wet) or IV NSCLC.
2. Patients with positive EGFR exon19 or exon21 mutation as confirmed by direct sequencing histologically.
3. Signing the informed consent form.
4. The vital organ functions are tolerant to therapy.
5. Have recovered to CTCAE2 grade below from toxicity of previous chemotherapy and radiotherapy
6. PS 0-2 scores.
7. Functional reserve of bone marrow is adequate, e.g. white blood cell count ≥3.0×10^9^/L, planet count ≥90×10^9^/L, and HB≥80×10^9^/L.
8. Serum bilirubin is 2 times less than the upper limit of normal (ULN), ALT and AST are 3 times less than ULN; for liver metastases, ALT and AST need to be 5 times less than ULN; creatinine should be 2 times less than ULN.

## 5.3 Exclusion criteria

1. Informed consent is not provided.
2. Women of pregnancy or breastfeeding.
3. Have difficulty in swallowing.

## 5.4 Drop-out criteria

In addition to the following conditions, the patients will persist to the end of study:

1. Safety issues as determined by investigators.
2. Serious noncompliance to the protocol as determined by investigators.
3. Enrollment by mistakes.

## 5.5 Withdrawal criteria

The following withdrawal is caused by noncompliance and treatment-unrelated reasons:

1. The patients receive other treatments than this protocol during the study;
2. The patients request to withdraw from this trial during the study;
3. The patients cannot further receive this protocol due to concomitant non-neoplastic diseases.
4. Disease progression.

## 5.6 Treatment of noncompliance and dropouts

Non-compliance and dropouts need to be followed up and reflected according to the principle of “intention-to treat (ITT)” in statistical analysis.

# 6. Endpoints

## 6.1 Primary endpoints

Remission rate and the time of PFS

## 6.2 Secondary endpoints

1. Time of OS

2. Occurrence of respective adverse events

3. Cost-effectiveness analysis

## 6.3 Exploration endpoints

For patients who are willing to provide their blood during treatment until disease progression and/or tumor samples at the time of disease progression, we will detect biomarkers correlated with EGFR pathway in plasma and/or tumor samples and monitor the dynamic changes in EGFR mutations.

# 7. Study Procedure

The physician in charge of screening should verify whether the patients are eligible before applying for enrollment. The patient will not participate in this trial if any of inclusion criteria is incompetent or any of exclusion criteria is conformed. Once participating in this study, the patient ID will be the unique identifier.

**Evaluation Schedule**

| Follow-up period | Screening | | Visit1 | Visit2 | Visit3 | Visit4 | Visit5, 6^e^… | Visit50 | Visit51、52 |
| --- | --- | --- | --- | --- | --- | --- | --- | --- | --- |
| Days | -21 | -14 | 1 | 7 days  (±3) | 37 days  (±3) | 97 days  (±3) | Every 2 months post V4  (±3) | 30days after discontinuation | Every 60 days (±3) |
| Informed consent | Ⅹ |  |  |  |  |  |  |  |  |
| Medical and surgical history ^a^/smoking history/ demographics/gene test ^a^ | Ⅹ |  |  |  |  |  |  |  |  |
| Physical examination/functional status/vital sign/height and weight |  | Ⅹ | Ⅹ | Ⅹ | Ⅹ | Ⅹ | Ⅹ | Ⅹ |  |
| Blood examination such as blood routine test and biochemistry test  ^b^ |  | Ⅹ | Ⅹ | Ⅹ | Ⅹ | Ⅹ | Ⅹ | Ⅹ |  |
| Pregnancy test (urine or blood) ^c^ |  | Ⅹ |  |  |  |  |  |  |  |
| Chest X-ray | Ⅹ |  |  | Ⅹ |  |  |  |  |  |
| CT-enhanced scan ^d^ | Ⅹ |  |  |  | Ⅹ | Ⅹ | Ⅹ |  |  |
| Erlotinib/Gefitinib |  |  | Ⅹ | Ⅹ | Ⅹ | Ⅹ | Ⅹ |  |  |
| Adverse reactions/adverse events/concomitant medicines ^d^ | Ⅹ |  | Ⅹ | Ⅹ | Ⅹ | Ⅹ | Ⅹ | Ⅹ |  |
| Relapse/survival data and anti-tumor therapy |  |  |  |  |  |  |  | Ⅹ | Ⅹ |
| Blood samples collection^h^ |  |  | Ⅹ | Ⅹ | Ⅹ | Ⅹ | Ⅹ |  |  |

X refers the scheduled test. The superscripts describe the specified tests or records.

1. Comprehensive medical and surgical history of all related diseases will be asked, especially previous and existing lung diseases.
2. The blood examinations consist of blood routine test, biochemistry test and CEA, e.g. anti-agglutination test.
3. Premenopausal women of childbearing potential must have the result of urine or blood pregnancy test 14 days before enrollment. The test needs to be repeated if pregnancy is suspected during the study,
4. Imaging examinations are performed routinely. The imaging examinations at screening are valid in 21 days, and the subsequent examinations must be completed within ±7 days as scheduled.
5. The following is performed by the same guidance as Visit 4.
6. The following is performed by the same guidance as Visit 5.
7. It is examined during clinical symptoms, as necessary or as suggested by the investigators.
8. Only for patients who are willing to provide their blood during. The last time to collect the blood sample is at the time of disease progression.

## 7.1 Subject screening and baseline measurements

Subjects who are eligible for inclusion criteria are screened by each test.

The objective of baseline measurement is to ensure the comparability of treatment group and control group. All baseline measurements must be completed before initiating the therapeutic regimens (see Table 2). The symptoms and vital signs of the patients and the results of all necessary examinations must be recorded in the registration form.

## 7.2 Therapeutic regimens

The recent efficacy evaluation is based on RECIST criteria and the toxicity assessment follows CTC criteria version 3. After the baseline measurements are completed before treatment, the subjects are randomly divided into erlotinib group or gefitinib group by 1:1. The safety data of all subjects will be evaluated at any time.

### 7.2.1 Erlotinib group

Randomized patients of erlotinib group will receive oral administration of erlotinib 150mg qd and take chest, blood, liver and kidney function and CEA examinations in 1 week after treatment followed by follow-ups in 1 month and 2 months later for efficacy assessment. In case of CR, PR, SR or clinical benefits, return visits will be performed every 3 months until the patients experience disease progression or withdraw from the study. Then, survival follow-ups will be conducted.

### 7.2.2 Gefitinib group

Randomized patients of gefitinib group will receive oral administration of gefitinib 250mg qd and take chest, blood, liver and kidney function and CEA examinations in 1 week after treatment followed by follow-ups in 1 month and 2 months later for efficacy assessment. In case of CR, PR, SR or clinical benefits, return visits will be performed every 3 months until the patients experience disease progression or withdraw from the study. Then, survival follow-ups will be conducted.

### 7.2.3 Auxiliary medication and treatments of common complications

In course of entire clinical trial, alleviative radiotherapy and bisphosphonate therapy are allowed. The acceptable drugs contain supportive symptomatic treatments such as dehydrant agents, drugs increasing leukocyte count, blood transfusion, platelet transfusion, antibiotics, antipyretic analgesics and anesthetics, however, the drug name, treatment of time and the reason of use should be recorded for these treatments. They will not be affected after disease progression.

### 7.2.4 Dosage adjustment due to drug toxicity

The doses need to be adjusted according to the package inserts of erlotinib and gefitinib and clinical routine. The subjects who fail to relieve to level 2 in 2 weeks after symptomatic treatments will discontinue the treatment but still be included in follow-up. The reasons of dosage adjustment or delay, the use of supportive therapy and the results should be recorded in CRF.

### 7.2.5 Reporting of serious adverse events

Regardless of whether it is related to the study treatment, any adverse events resulting in the following conditions after enrollment should be reported to Ethics Committee, the sponsor (CRA) and FDA within 24 hours when it is occurred:

- Death
- Hospitalization or prolonged hospitalization
- Life-threatening events (have death risk immediately)
- Permanent or serious disability/incapability

The specified clinical outcome, death (due to disease progression), will be reported as serious adverse event only when the investigators believe the death is related to the study treatment. Any events due to disease progression will be not regarded as serious adverse events.

## 7.3 Follow-up procedure

All patients should undergo follow-up regardless that the enrolled patients drop out or withdraw from this study for any reason.

The follow-up of withdrawal: the reason of withdrawal should be explained. The patients need to continue the follow-up until the end of study after withdrawal from the study. In this case, the tumor condition, the functional status and the following therapeutic regimens should be recorded as well.

### 7.3.1 Schedule

The subjects will undergo a follow-up in 1 week after enrollment and 1 month and 2 months later; the follow-up will be performed every 3 months after it is confirmed effective. Survival follow-up will be conducted by phone after disease progression.

### 7.3.2 Follow-up plan

Physical examination, blood test and tumor biomarker examination, and imaging examination will be performed routinely.

### 7.3.3 Follow-up record

1. Follow-up data
2. Efficacy evaluation of TKI and subsequent periodic follow-up assessments (RECIST criteria, see the attachment)
3. Primary lesion CR; PR; SD; PD
4. PS score: ECOG scoring standard 0~4
5. Physical examination: record vital signs and abnormal conditions
6. Adverse events: time of occurrence and termination, grade (CTC 3.0 criteria, see the attachment)
7. Concomitant medication: drug name, dosage, usage, time of initiation and termination, reason of use
8. Disease progression (or occurrence of symptomatic brain metastasis): time, location; post-progression therapy
9. Survival
10. Date of death
11. Reason of death: die of this disease, secondary tumor, non-tumor, complication, treatment toxicity or unclear reason.

# 8. Efficacy and Safety Evaluations

This study mainly performs treatment intervention using erlotinib / gefitinib in patients with exon21 mutation. The primary endpoint is the remission rate and the effect to prolong survival in patients with exon21 mutation.

## 8.1 Efficacy assessment

The patients will undergo efficacy assessment at Visit 2 after enrollment and every 3 months later:

1. Remission rate
2. PFS time
3. Overall survival

## 8.2 Adverse reactions

The subjective and objective symptoms are recorded according to “CTC3.0 solid tumor toxicity grading criteria”. The adverse reactions described in this evaluation criteria are recorded by severity (Grade 0-4). The side effects that are not mentioned in the standard are recorded by mild (+), moderate (++), moderate-severe (+++) and severe (++++), and the details of side effects should be recorded in the remarks of case report form to ensure the side effects will be evaluated consistently in the future. (30 days after discontinuation, original or new treatment-related AE as determined by the investigators needs to be followed up until the subject is recorded or died, and SAE should be reported as required.)

## 8.3 PS score

PS scores follow ECOG (Zubrod) scoring criteria.

# 9. Ethical Requirements

This study will be performed in compliance with Helsinki Declaration and existing Good Clinical Practice (GCP). As required by GCP, this study must be implemented after the medical ethics committee of the study site approves. The principal investigator needs to participate in GCP training of this study and be responsible for quality of this clinical trial.

The investigators should explain any relevant questions to the patients to be enrolled to eliminate their misgivings about participation in this study. All participants have to sign the informed consent form before enrollment. The patients have rights to withdraw from this clinical trial at any time. After the subject withdraws from the study, the investigators should provide treatment-related consultation for the patients so that they can continue to receive appropriate treatments.

# 10. Study Quality Control

1. Prior to the study, the principal investigator and the investigators of this site should learn GCP principle and discuss this protocol in order to fully understand the requirements and the procedure of this study.
2. The principal investigator and the investigators of this site should assure the study quality, control the inclusion and exclusion criteria strictly, and fill in CRF carefully. Correcting wrong data can be deleted by a single oblique line followed by writing correct number nearby with signature and date. The original amendment should be identifiable. The principal investigator should inspect every CRF carefully and sign after it is confirmed complete.
3. The medical history is required to be real and reliable and be maintained appropriately for selective examination at any time.

# 11. Data Acquisition and Analytical Methods

## 11.1 Data acquisition

1. All data should be recorded in the Case Report Form (CRF) or CRF software timely, truly and in detail. CRF will be considered valid when they are filled by specific persons assigned by the study site with the signature of the principal investigator.
2. After the clinical trial is completed, the study site will organize all forms and compile a final report as required.

## 11.2 Statistical analysis

In this study, SPSS17.0 software will be used for data analysis. Quantitative endpoints are described by median, minimum and maximum, qualitative endpoints are expressed by frequency and percentage. The general demographic characteristics of subjects are shown by descriptive analysis; chi-square test and Fisher's exact test are used to compare the equivalence of demographic characteristics, efficacy evaluation and occurrence of adverse events between both groups; log-rank test is used to analyze the disease progression and survival of subjects; COX regression multivariate analysis is used to analyze the prognostic factors for disease progression and survival of subjects. All statistical tests are performed two-sided, and a statistically significant difference is considered when P value is less than or equal to 0.05.

## 11.3 Mid-term analysis

After 70 eligible subjects are enrolled in this randomized, controlled phase II clinical trial and the 70th subject completes 12-month follow-up, a mid-term analysis will be performed as follows:

- Number of enrolled patients and time to be completed;
- Number of compliance and non-compliance to the therapeutic regimens;
- Evaluate the quality of data based on the time when submitting the data, and the integrity and the accuracy;
- Occurrence and grading of toxic and side effects.

Based on the above data, the central office will summarize and report at the study site meeting periodically. If necessary, the protocol may be revised or terminated.

# References

1. Thatcher N, Chang A, Parikh P et al: Gefitinib plus best supportive care in previously treated patients with refractory advanced non-small-cell lung cancer: Results from a randomised, placebo-controlled, multicentre study (Iressa Survival Evaluation in Lung Cancer). Lancet 366:1527–1537, 2005

2. Shepherd FA, Rodrigues Pereira J, Ciuleanu T, et al: Erlotinib in previously treated non-small-cell lung cancer. N Engl J Med 353:123–132, 2005

3. Fukuoka M, Yano S, Giaccone G, et al: Multiinstitutional randomized phase II trial of gefitinib for previously treated patients with advanced non-small-cell lung cancer (the IDEAL 1 trial). J Clin Oncol 21:2237–2246, 2003

4. Giaccone G, Herbst RS, Manegold C, et al: Gefitinib in combination with gemcitabine and cisplatin in advanced non-small-cell lung cancer: A phase III trial—INTACT 1. J Clin Oncol 22:777–784, 2004

5. Paez JG, J¨anne PA, Lee JC, et al: EGFR mutations in lung cancer: Correlation with clinical response to gefitinib therapy. Science 304:1497–1500, 2004

6. Lynch TJ, Bell DW, Sordella R, et al: Activating mutations in the epidermal growth factor receptor underlying responsiveness of non-small-cell lung cancer to gefitinib. N Engl J Med 350:2129–2139, 2004

7. Bell DW, Lynch TJ, Haserlat SM, et al. Epidermal growth factor receptor mutations and gene amplification in non-small-cell lung cancer: molecular analysis of the IDEAL/INTACT gefitinib trials. J Clin Oncol. 2005,23(31):8081-92.

8. Mok T, Wu Y-L, Thongprasert S, et al: Phase III, randomised, open-label, first-line study of gefitinib (G) vs carboplatin/paclitaxel (C/P) in clinically selected patients (PTS) with advanced non-small-cell lung cancer (NSCLC) (IPASS). Ann Oncol 19 (S8): viii1- viii4, 2008 (suppl 8)

9. Jackman DM, Yeap BY, Sequist LV, et al: Exon 19 deletion mutations of epidermal growth factor receptor are associated with prolonged survival in non-small cell lung cancer patients treated with gefitinib or erlotinib. Clin Cancer Res 12: 3908-3914, 2006

10. Riely GJ, Pao W, Pham DK, et al: Clinical course of patients with non-small cell lung cancer and epidermal growth factor receptor exon 19 and exon 21 mutations treated with gefitinib or erlotinib. Clin Cancer Res 12: 839-8444, 2006

11. Massuti B, Morán T, Porta R, et al: Multicenter prospective trial of customized erlotinib for advanced non-small cell lung cancer (NSCLC) patients (p) with epidermal growth factor receptor (EGFR) mutations: Final results of the Spanish Lung Cancer Group (SLCG) trial. J Clin Oncol 27:15s, 2009 (suppl; abstr 8023)

12.Yang CH, Yu CJ, Shih JY, et al. Specific EGFR Mutations Predict Treatment Outcome of Stage IIIB/IV Patients With Chemotherapy-Naïve Non–Small-Cell Lung Cancer Receiving First-Line Gefitinib Monotherapy. J Clin Oncol 26(16): 2745-2753, 2008

13. Fukuoka M, Wu Y, Thongprasert S, et al. Biomarker analyses from a phase III, randomized, open-label, first-line study of gefitinib (G) versus carboplatin/paclitaxel (C/P) in clinically selected patients (pts) with advanced non-small cell lung cancer (NSCLC) in Asia (IPASS). J Clin Oncol 27:15s, 2009 (suppl; abstr 8006).

14. Costa DB, Schumer ST, Tenen DG, et al. Differential responses to erlotinib in epidermal growth factor receptor (EGFR)-mutated lung cancers with acquired resistance to gefitinib carrying the L747S or T790M secondary mutations. J Clin Oncol. 2008,26(7):1182-4.
